# β_1_-Blockers Enhance Inotropy of Endogenous Catecholamines in Chronic Heart Failure

**DOI:** 10.3389/fcvm.2021.639562

**Published:** 2021-06-04

**Authors:** Thomas J. Feuerstein, Eberhard Schlicker

**Affiliations:** ^1^Sektion für Neuroelektronische Systeme, Klinik für Neurochirurgie, Universität Freiburg, Breisgau, Germany; ^2^Freiburg Institute for Advanced Studies, Albert-Ludwigs-Universität Freiburgf and Institut für Pharmakologie und Toxikologie, Universität Bonn, Bonn, Germany; ^3^Institut für Pharmakologie und Toxikologie, Universität Bonn, Bonn, Germany

**Keywords:** chronic heart failure, sympathetic tone, homodimer, negative cooperativity, binomial distribution, receptor reserve

## Abstract

Although β_1_-blockers impressively reduce mortality in chronic heart failure (CHF), there are concerns about negative inotropic effects and worsening of hemodynamics in acute decompensated heart failure. May receptor theory dispel these concerns and confirm clinical practice to use β_1_-blockers? In CHF, concentrations of catecholamines at the β_1_-adrenoceptors usually exceed their dissociation constants (*K*_D_s). The homodimeric β_1_-adrenoceptors have a receptor reserve and display negative cooperativity. We considered the binomial distribution of occupied receptor dimers with respect to the interaction of an exogenous β_1_-blocker and elevated endogenous agonist concentrations > [*K*_D_s], corresponding to an elevated sympathetic tone. Modeling based on binomial distribution suggests that despite the presence of a low concentration of the antagonist, the activation of the dimer receptors is higher than that in its absence. Obviously, the antagonist improves the ratio of the dimer receptors with only single agonist activation compared with the dimer receptors with double activation. This leads to increased positive inotropic effects of endogenous catecholamines due to a β_1_-blocker. To understand the positive inotropic sequels of β_1_-blockers in CHF is clinically relevant. This article may help to eliminate the skepticism of clinicians about the use of β_1_-blockers because of their supposed negative inotropic effect, since, on the contrary, a positive inotropic effect can be expected for receptor-theoretical reasons.

## Introduction

β-Adrenoceptors (β-AR)s are activated by the catecholamines noradrenaline (NA) and adrenaline (A), and are members of the adrenoceptor family of the seven-transmembrane superfamily of receptors. There are three β-AR subtypes: β_1_, β_2_, and β_3_. Activation of adenylate cyclase through the guanine nucleotide-binding regulatory protein G_S_ is the classic, but not the only, mechanism of β-AR action (1). Cardiac β_1_-ARs increase the heart rate, myocardial contractility, impulse conduction, and pacemaker activity. They represent 75–80% of the total β-AR density in human adult cardiac myocytes (2). β_2_-ARs (3–5) comprise about 15–18%, and the remaining 2–3% is β_3_-AR (2).

β_1_-Blockers improve the hemodynamic situation of chronic heart failure (CHF) (6), and some of them even increase survival (6–8). Mortality in CHF is reduced by about 34% (9). However, the clinically beneficial β_1_-AR antagonism in CHF has long been doubted. A leading textbook has stated still in 1996 that it is unclear whether β-blockers improve survival in heart failure patients (10). Apart from the unavailability of large clinical studies at that time, one reason for the skepticism against the use of β-blockers in CHF may have been the absence of a convincing, pathophysiologically founded, rationale for this use.

In the meantime, some β-blockers have become a standard strategy to increase survival in CHF. Guidelines suggest the use of bisoprolol, carvedilol, metoprolol succinate, and nebivolol for this purpose (6–8). The latter is effective in patients >70 years only, and a positive effect has been shown for the combined endpoint of survival or hospitalizations only (11). The former three drugs had similar effects on all-cause mortality among patients with CHF in a study on 6,010 outpatients with stable CHF and a reduced left ventricular ejection fraction (12).

The beneficial effect of β_1_-blockers is mainly ascribed to the following conditions:

- Negative chronotropy; among others, they reduce the incidence of atrial fibrillation and control ventricular rate (7).
- They protect the heart against left ventricular remodeling elicited by endogenous catecholamines via β_1_-adrenoceptors (13, 14).
- They protect the heart indirectly via inhibition of the renin–angiotensin cascade (15).
- β_1_-Blockers antagonize anti-β_1_-AR antibodies constitutively stimulating β_1_-ARs (16).
- Moreover, they lead to an induced sympatholysis (2).

The therapeutic rationale to use β_1_-blockers in CHF is not easy to understand and has been considered a contradiction for many years (11). One reason, for instance, is the doubtfulness whether the abovementioned negative chronotropic effect can compensate for the simultaneous negative inotropic effect to be expected, at first glance, under β_1_-AR blockade (11). The well-known β_1_-AR downregulation in CHF (17) suggests that β_1_-ARs play a major role in the development of CHF.

Here, we provide evidence that *positive, not negative*, inotropic effects may occur in CHF patients treated with a low concentration of a β_1_-AR antagonist.

## Methods

To understand how the binomial distribution solves the riddle of negative inotropic β_1_-blockers inducing positive inotropic effects, two fundamental properties of β_1_-ARs in the human heart must be recalled.

First, β_1_-ARs have a receptor reserve (spare receptors) (18–20); in other words, the concentrations of catecholamines (the endogenous agonists at β_1_-ARs) that produce the half-maximum effects (*EC*_50_s) are lower than their dissociation constants *K*_D_s at the β_1_-ARs. Note that during CHF, the concentration of the endogenous agonists at the β_1_-AR may even exceed their *K*_D_s (21–23). For the sake of simplicity, the endogenous catecholamines are called endogenous agonist below with a single *K*_D_ only.

Second, β_1_-ARs may occur as homodimers (24, 25). To activate G proteins, a pentameric structure constituted of one GPCR homodimer and one heterotrimeric G protein may provide the main functional unit (26). The colocalization between two β_1_-AR particles is transient; dimerization increases with receptor density (24). However, the dimers are stable over a 10-fold range of receptor expression levels (27). Agonist stimulation does neither alter receptor dimerization nor lateral mobility within the cellular membrane (24). It is the TM5 interface of the ligand-free β_1_-AR that can partner with TM4 or TM6, depending on the conformation of the dimeric receptors. However, an agonist-induced receptor–G protein interaction depends on rearrangements of TM5 and TM6 within the seven-helical domain bundle, which may suggest that dimerization occurs between the partners TM4 and TM5, leaving TM6 for G protein interaction behind (26).

In addition, agonist activation of one subunit (protomer) is sufficient to induce the inotropic response obtained from this dimer: Ligand occupancy to the first protomer is enough to produce a significant G protein activation and functional response (26), and the binding of the endogenous agonist to the second subunit is negatively influenced after the first one has been occupied [negative cooperativity, (25)]. Here, negative cooperativity means that the binding of an agonist to the first protomer decreases the affinity of the agonist for the second one: Both protomers of a homodimer contribute to the allosteric modulation, and the same ligand is the allosteric modulator (binding to the first protomer) and the modulated target (binding to the second protomer). This may result in two different levels of ligand-mediated signaling that would depend on the concentration of the ligand. Thus, negative cooperativity could provide a mechanism that protects the biologic system against acute elevations of an endogenous agonist (26).

### The Binomial Distribution Describes the Occupancy of the Dimeric β_1_-Spare Receptors

The total number of receptors per dimer is *n* = 2. The number of occupied receptors is *i* = 0, 1, or 2; *i* = 1 is the (minimal) number of β_1_-ARs of a dimer that must be activated to obtain a maximum agonist effect of this dimer. Thus, half of the dimer receptors are spare receptors.

If 0 < *i* ≤ *n*, or 0 < 1, 2 ≤ 2, the number *i* of occupied receptors has a binomial distribution B(*n, q*) with parameters *n* = 2 and *q* (Box 1).

Box 1 **A Short Introduction Into Binomial Distribution**

The *binomial distribution* indicates the number of successes in a sequence of *n* independent experiments, each asking a *yes*–*no* question. In our context, *yes* is a single binding success to a receptor dimer; *no* means absence of binding. A receptor dimer only allows *n* = 2 independent experiments. The first experiment refers to the first receptor, the second to the second receptor, to be occupied or not. The binding success at each receptor has the probability *q*. The binomial distribution, B(*n, q*), equals $\left(\begin{array}{c} n\\ i \end{array}\right)$ *q*^*i*^(1 − *q*)^*n*−*i*^. $\left(\begin{array}{c} n\\ i \end{array}\right)=\;\frac{n!}{i!\left(n-i\right)!}$; thus, for *i* = 0, 1, 2 and *n* = 2, $(\begin{array}{c} 2\\ 0 \end{array})$ = 1, $\left(\begin{array}{c} 2\\ 1 \end{array}\right)$ = 2, and $\left(\begin{array}{c} 2\\ 2 \end{array}\right)$ = 1. *i* successes occur with probability *q*^*i*^, and *n* – *i* failures occur with probability (1 – *q*)^*n* − *i*^. However, the *i* successes can occur anywhere among the two trials, and there are $\left(\begin{array}{c} 2\\ i \end{array}\right)$ different ways of distributing *i* successes in a sequence of two trials: One way only to distribute the absence of occupations on both dimer receptors, two ways for occupying the first and leaving the second free or leaving the first free and occupying the second, again only one way to distribute two occupations among two receptors.

*Example*: Let us say you roll six times with a normal die. What is the probability that the 6 will be rolled four times? The answer is $\left(\begin{array}{c} 6\\ 4 \end{array}\right){\left(\frac{1}{6}\right)}^{4}{\left(1-\frac{1}{6}\right)}^{6-4}=0.008$. Thus, this probability is 0.8%.

**Figure 1:**
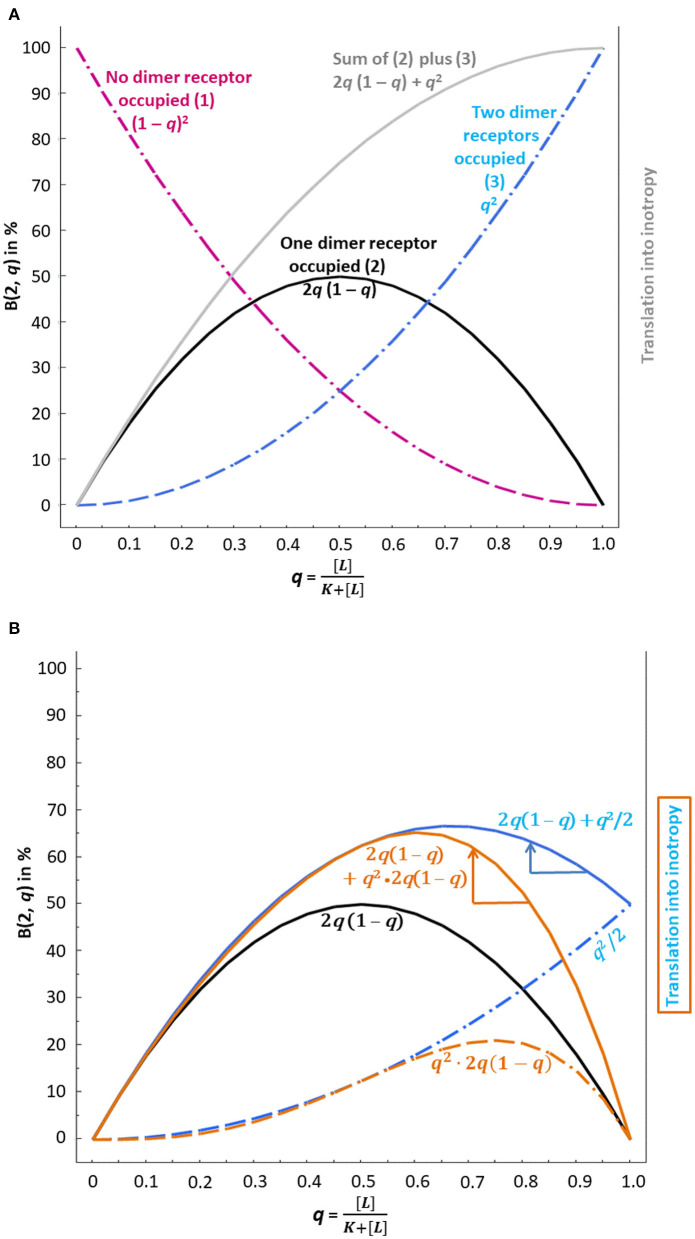
Binomial distribution and possible translation into inotropy of β_1_-AR ligand binding at dimer receptors *without consideration of negative cooperativity* **(A)** and *with consideration of negative cooperativity* **(B)**. Usually, *concentration*–response curves with lg *concentrations* on the abscissa are used; here the x-axis is characterized by the probability *q* of binding instead. “*q*,” i.e., the fractional receptor occupation $\frac{\left[L\right]}{K+\left[L\right]}$, corresponds to the relative frequency of binding, which depends on the concentration of the ligand [*L*]. Ligand binding, i.e., the agonist and its interaction with the antagonist, then translates into inotropy according to the binomial distribution. **(A)** The dashed purple curve, which indicates the probability that no dimer receptor is occupied, does – of course – not translate into inotropy. The black and the dashed blue curves, however, reflect the presence of an agonist and, therefore, translate into inotropy. The gray curve is the sum of the black and the dashed blue curve and reflects a 50% receptor reserve since the activation of one of two dimer receptors yields the same maximum effect (at *q* = 1) as the activation of two receptors. **(B)** The presence of an antagonist changes the inotropic effect of agonist binding. *q*^2^/2 has been used to model the fact that binding to the first receptor is already sufficient to obtain the maximum response, whereas the second receptor does not contribute any further (dashed blue curve without antagonist interaction). The latter curve and the curve for activation of one receptor only (black curve) add to the solid blue curve. Now the antagonist effect comes into play. The blue arrow shows that at *q* > 0.67, the β_1_-AR antagonist – by shifting the curve to the left (see blue arrow) – can increase inotropy. Another modeling of negative cooperativity reflects the condition that the functionally relevant first binding event extends to all dimers covered by the condition “both dimer receptors occupied,” which leads to *q*^2^ · 2*q*(1 – *q*) (dashed brown curve). The latter curve and the curve for activation of one receptor only (black curve) add to the solid brown curve. Again, the antagonist effect comes into play here. The brown arrow indicates the extent of possible inotropy increase. An increase in inotropy is only possible at *q* > 0.61.

For *i* = 0, no receptor of the dimer is occupied; for *i* = 1 or 2, one or two receptors are occupied, respectively. “*q*” is the fractional receptor occupation, $\frac{\left[L\right]}{K+\left[L\right]}\;$, with *L* representing the β_1_-AR ligand (agonist or antagonist), [*L*] its concentration, and *K* the dissociation constant of the ligand (*K* = *K*_D_ for an agonist, *K* = *K*_A_ for an antagonist). Figure 1 represents diagrams with $\frac{\left[L\right]}{K+\left[L\right]}\;$ on the x-axis and B(2, *q*) on the y-axis.

### The Binomial Occupancy May Be Exemplified by a Pure Antagonist as Ligand of β_1_-ARs

*K*_A_ of a pure (neutral) β_1_-blocker is 10^−7^ M [e.g., metoprolol, (22)]. Using this drug at a concentration of 10^−7^ M then yields $q=\frac{\left[K_{a}\right]}{K_{a}+\left[K_{a}\right]}=0.5$, and the following distributions ensue:

No receptor occupied: With $\left(\begin{array}{c} n\\ i \end{array}\right)$ *q*^*i*^(1 − *q*)^*n*−*i*^, the probability that no receptor is occupied (*n* = 2 and *i* = 0) is 25%; $\left(\begin{array}{c} 2\\ 0 \end{array}\right)$ 0.5^0^(1 − 0.5)^2−0^ = 1 · 1 · (0.5)^2^ = 0.25 according to [$\left(\begin{array}{c} 2\\ 0 \end{array}\right)$ *q*^0^(1 − *q*)^2−0^ = (1 – *q*)^2^].

Thus, 25% of all dimers have no blocked receptor and can be activated by an endogenous β_1_-AR agonist occupying at least one receptor of each dimer (see Figure 1A, dashed purple curve, where $\frac{\left[L\right]}{K+\left[L\right]}=0.5$ on the x-axis corresponds to 25% on the y-axis).

One β_1_-AR blocked and one non-occupied: 50%.

$\left(\begin{array}{c} 2\\ 1 \end{array}\right)$ 0.5^1^(1 − 0.5)^2−1^ = 2 · 0.5 · 0.5 = 0.5 according to [$\left(\begin{array}{c} 2\\ 1 \end{array}\right)$ *q*^1^(1 − *q*)^2−1^ = 2 · *q* · (1 – *q*)] (see Figure 1A, black curve, where $\frac{\left[L\right]}{K+\left[L\right]}=0.5$ on the x-axis corresponds to 50% on the y-axis).

Both β_1_-ARs blocked: 25%; these are not accessible for agonists.

$\left(\begin{array}{c} 2\\ 2 \end{array}\right)$ 0.5^2^(1 − 0.5)^2−2^ = 1 · 0.25 · 1 = 0.25 according to [$\left(\begin{array}{c} 2\\ 2 \end{array}\right)$ *q*^2^(1 − *q*)^2−2^ = *q*^2^] (see Figure 1A, dashed blue curve, where $\frac{\left[L\right]}{K+\left[L\right]}=0.5$ on the x-axis corresponds to 25% on the y-axis).

All calculations and the creation of Figure 1 have been performed with *JMP*® 10 (SAS Institute, Heidelberg, Germany).

## Results and Discussion

How can we now explain that the presence of a pure (neutral) β_1_-AR antagonist enhances the action of the endogenous agonist? This will be discussed below, using a *K*_*D*_ value for the endogenous β_1_-AR agonist of 10^−8^ M [(28), see also Box 2] and a *K*_A_ value for the pure antagonist of 10^−7^ M (see above).

Box 2 **Searching for the *K*_D_ value of NA and A**

- The plasma concentrations of the endogenous catecholamines, [NA] and [A], measured in controls and heart transplant recipients (28) were in the low nanomolar range (Table below). Considering the catecholamine concentrations of 487 and 2,599 ng/L in both groups, Ferretti et al. (28) obtained a β_1_-adrenoceptor affinity constant for the catecholamines of ~487/176.2 ng/L = 2.76 nM for the controls and ~2,599/176.2 ng/L = 14.75 nM for heart transplant recipients (with 176.2 being the mean of the molecular weights of NA and A) (Table). Thus, our assumed *K*_D_ of 10 nM for the endogenous agonists seems realistic, at least in the light of the available literature on the human heart.
- The paper by Baker (29) yielded *micromolar K*_D_s, i.e., ~100-fold lower *EC*_50_s for NA and A than assumed, suggesting a very high receptor reserve (Table). These values were obtained in binding experiments in whole CHO cells stably expressing human β_1_-adrenoceptors resembling the low-affinity sites detected in membranes in the presence of GTP. For comparison, Hoffmann et al. (22) studied binding affinities of agonists in the presence of 100 μM GTP. This non-physiological addition again yielded micromolar *K*_I_s of NA and A, which, however, do not display the real *K*_D_s of the endogenous agonists at physiological β_1_-adrenoceptors.
- By the way, an extremely high receptor reserve, which has to explain a factor of ~100 between *EC*_50_ and *K*_D_ is highly unlikely: One has to emphasize that the classical calculation of concentration–response curves of spare receptors according to the operational model of Black and Leff (30) is misleading [see, for instance, (31–33)]. Thus, we do not assume such a high β_1_-adrenoceptor reserve in the human heart. In addition, the affinity shift between the β_1_-adrenoceptor-rich controls and the β_1_-adrenoceptor-poorer heart transplant recipients, corresponding to *Lg* (14.75/2.76) = 0.73 *Lg*-units, would correspond to a decrease in spare receptors from about 75% to 25%; see Figure 4 of Feuerstein et al. (34). The assumed receptor reserve of 50% in the manuscript complies with this order of magnitude.

**Table:** 

			Catecholamine concentration (nM) β_1_-adrenoceptor affinity constant (*K*_D_, nM)^*^ β_1_-adrenoceptor potency (*EC*_50_, nM)^°^	
			**Noradrenaline**	**Adrenaline**
*In vivo*	Controls	Ferretti et al. (28)	1.63	0.55
			2.76^⋆^	
	Heart transplant recipients		8.31	1.48
			14.75^⋆^	
*In vitro*	CHO cells	Baker (29)	1,820^⋆^	7,080^⋆^
			11.48°	24.55°
*In vitro*	CHO cells	Hoffmann et al. (22)	3,570^⋆^	3,970^⋆^

Let us assume that 3,570 nM would be the correct *K*_D_ for NA and that 11.48 nM would be the correct *EC*_50_. Thus, a corresponding spare receptor-induced shift was *Lg* (3,570/11.48) = 2.49 *Lg*-units. A translation of this shift into a degree of receptor reserve according to Feuerstein et al. (34) estimates 1,000 β_1_-adrenoceptors per heart myocyte, being the so-called functional unit, and 999 spare receptors. Thus, the activation of 1 of 1,000 adrenoceptors per myocyte would suffice to induce a maximum inotropic response. Thus, linking a binding *K*_D_ with a functional *EC*_50_ seems unrealistic.

### Occupancy by the Endogenous β_1_-AR Agonist Alone or in the Presence of the Pure Antagonist

In the *absence of pure antagonist*, the term $q=\frac{\left[endogenous\;agonist\right]}{10^{-8}\;+\;\left[endogenous\;agonist\right]}$ will be, at [endogenous agonist] = 10^−7^ M, $\frac{\left[10^{-7}\right]}{10^{-8}\;+\;[10^{-7}]}=0.91.$ This means that 91% of β_1_-ARs of all dimers are occupied by the endogenous β_1_-AR agonist, either by only one agonist molecule (yielding $\left(\begin{array}{c} 2\\ 1 \end{array}\right)$ 0.91^1^(1 − 0.91)^2−1^ = 16.4%, see Figure 1A, black curve at *q* = 0.91) or by two agonist molecules [yielding $\left(\begin{array}{c} 2\\ 2 \end{array}\right)$ 0.91^2^(1 − 0.91)^2−2^ = 82.8%, see Figure 1A, dashed blue curve at *q* = 0.91]. The sum of both the black curve, depicting the condition “one of two receptors is occupied” and the dashed blue curve, depicting the condition “two of two receptors are occupied” yields the gray curve in Figure 1A. This sum corresponds to a receptor reserve of 50% since the activation of one receptor leads to the same maximum effect from the respective dimer as does the activation of both dimer receptors. This assumed amount of receptor reserve of 50% only in the human heart corresponds to the findings of Brown et al. (19) who stated that in the human heart, receptor reserve was rather low and declined further with an increasing degree of heart failure (see also Box 2). The two conditions mentioned, represented by the gray curve in Figure 1A, will be translated into inotropy when the signal of agonist occupation of one or two dimer receptors is transduced.

The *presence of 10*^−7^ *M of the pure antagonist* increases the agonist *K*_D_ with $q=\frac{\left[endogenous\;agonist\right]}{\;10^{-8}\;+\;10^{-7-8+7}\;+\;\left[endogenous\;agonist\right]}$. For explanation, the sum of exponents in 10^−7−8+7^ in the denominator corresponds to $10^{Lg[endogenous\;agonist]\;+Lg[K_{D}]-Lg[K_{A}]}$ with *Lg* = *log*_10_; see equation 1 in Mantovani et al. (35), which is based on the Cheng and Prusoff (36) equation. Thus, at 10^−7^ M, the antagonist doubles the endogenous agonist *K*_D_ of 10^−8^ M, thereby diminishing *q*. There is no other influence of the antagonist on the curves of Figure 1 than to increase the agonist *K*_D_, i.e., to shift *q* to the left.

### Receptor Theory Finally Translates Agonist Occupation of Dimer Receptors Into Inotropy

The probabilities that, at different agonist and antagonist concentrations, no receptor, one of two receptors, or both receptors of the dimers are activated can be calculated. Table 1 displays agonist binding probabilities at the dimer receptors in the absence and presence of the β_1_-AR antagonist administered at a concentration (10^−7^ M) that equals its *K*_*A*_ value (note that in Table 1, agonist concentrations are given in μM for better distinctness).

**Table 1:** Probabilities for agonist activations of one of two dimer receptors and of both of two dimer receptors, without assumed affinity reduction due to negative cooperativity [gray curve of Figure 1A; (a)], corresponding probabilities considering negative cooperativity as compression of the dashed blue curve of Figure 1A by 50% [solid blue curve of Figure 1B; (b)], and considering negative cooperativity as a product of the dashed blue curve and the black curve of Figure 1A [solid brown curve of Figure 1B; (c)] (K_D_ of endogenous agonist 10^−8^ M; K_A_ of antagonist 10^−7^ M).

	**a**		**b**			**c**		
	**Endogenous agonist alone**	**Endogenous agonist, presence of antagonist**	**Endogenous agonist alone**		**Endogenous agonist, presence of antagonist**	**Endogenous agonist alone**		**Endogenous agonist, presence of antagonist**
	***P* (1 and 2 of 2 occupied)%**	***P* (1 and 2 of 2 occupied)%**	***P* (1 and 2 of 2 occupied)%**		***P* (1 and 2 of 2 occupied)%**	***P* (1 and 2 of 2 occupied)%**		***P* (1 and 2 of 2 occupied)%**
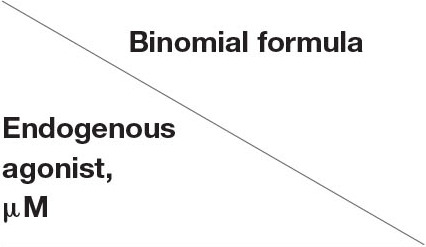	**2q(1 − q) + q^2^, gray curve in Figure 1A**		**2q(1 − q) + $\frac{{\text{q}}^{2}}{2}$, solid blue curve in Figure 1B**			**2q(1 − q)+ q^2^·2q(1 − q), solid brown curve in Figure 1B**		
0.00032	6.04	3.09	5.99		3.08	5.95		3.07
0.001	17.36	9.30	16.94		9.18	16.67		9.09
0.0032	42.28	25.44	39.39		24.51	38.61		24.02
0.01	75.00	55.56	62.50		50.00	62.50		49.38
0.032	94.23	84.99	65.37	<	66.23	57.58	<	65.28
0.1	99.17	97.22	57.85	<	62.50	30.19	<	47.07
0.32	99.91	99.65	52.92	<	55.42	11.53	<	21.09
1	99.99	99.96	50.98	<	51.90	3.88	<	7.54
3.2	100.0	100.0	50.31	<	50.62	1.25	<	2.48
10	100.0	100.0	50.10	<	50.20	0.40	<	0.80
32	100.0	100.0	50.03	<	50.06	0.13	<	0.25

Agonist binding to at least one dimer receptor is the basis of the translation of agonist occupation into inotropy. If one considers a simplified scenario (negative cooperativity neglected), the gray curve of Figure 1A yields the translation into inotropy as calculated in Table 1a. The presence of an antagonist decreases the activation probability at any agonist concentration since the blue curve is rising steadily. The absence of a maximum followed by a declining part of this blue curve excludes that the antagonist can lead to a higher level of the fractional receptor occupation *q* on the y-axis. In other words, without negative cooperativity between the dimer receptors, a β_1_-blocker cannot induce a positive inotropic effect.

### Positive Inotropy in the Presence of a β_1_-Blocker Requires Negative Cooperativity

Obviously, negative cooperativity has to be taken into account, i.e., that binding of one β_1_-AR agonist molecule to the first dimer receptor, which already induces the maximum inotropic response from this dimer, reduces the binding of the second agonist molecule to the partner receptor. The question is only how this negative cooperativity in the case of “two of two receptors occupied” can be modeled in the binomial distribution of dimer receptor binding. How must the dashed blue curve of Figure 1A be changed to reflect negative cooperativity?

### Possible Modeling of Negative Cooperativity

The binding reduction of the second agonist molecule diminishes the overall dimeric binding, i.e., at *q* = 1 on the x-axis, *q*^2^ (meaning that both β_1_-ARs are occupied) on the y-axis must be lower. Negative cooperativity may be expressed, for instance, by *q*^2^/2 since agonist binding to the first dimer receptor is already sufficient to obtain the maximum response and since binding to the second receptor does not contribute to the response. The dashed blue curve in Figure 1B represents these probabilities of *q*^2^/2. In this case, the sum of the dashed blue curve and of the black curve, i.e., the solid blue curve in Figure 1B, has a clear maximum at about *q* = 0.67. At this *q* value, the agonist concentration is 2.03 ^*^ [*K*_D_] (*q* = 0.67 = $\frac{\left[0.0203\right]}{\left[0.01]+[0.0203\right]}$). At agonist concentrations higher than 2.03 ^*^ [*K*_D_], the inotropic efficiency decreases again. Note that an antagonist-induced shift of *q* to the left at *q* > 0.67 on the x-axis *increases* the corresponding y-value.

By contrast, if the β_1_-blocker is used at a concentration that reduces the fractional receptor occupation to *q* < 0.67, an increase in positive inotropy is impossible.

Table 1b contains the beneficial condition. At an antagonist concentration equaling the *K*_A_, the corresponding inotropies may be increased. Thus, for concentrations of the endogenous agonist ≥0.032 μM [being larger than (*K*_D_) and corresponding to an elevated sympathetic tone], the agonist occupation in the presence of the antagonist is higher than that in its absence (see < signs).

Other modelings of negative cooperativity than *q*^2^ → *q*^2^/2 in the case of “two of two receptors occupied” are conceivable. In any case, negative cooperativity in the B(2, *q*)-curve must be represented by a declining part after a maximum. One additional modeling possibility may be:

Since the first binding event within each dimer already induces the maximum inotropic response, this relevant first binding event may extend to all dimers not yet covered by the conditions “no dimer receptor occupied” and “one of two dimer receptors occupied” {this complementary quantity corresponds to 1 – [(1 – *q*)^2^ + 2*q*(1 – *q*)] = *q*^2^}. It is the same as “both dimer receptors occupied” [corresponding to $\left(\begin{array}{c} 2\\ 2 \end{array}\right)$*q*^2^(1 − *q*)^2−2^ = *q*^2^]. These remaining dimers (to be equally occupied, but transducing differently due to negative cooperativity) then react to the endogenous agonist like those represented by the black curve in Figures 1A or 1B; the black curve is characterized by 2*q* (1 – *q*). To adjust for negative cooperativity, the term for the remaining dimers, *q*^2^, is multiplied by the term for “one of two dimer receptors occupied,” yielding *q*^2^ → *q*^2^ · 2*q* (1 – *q*). Note that also the first modeling corresponds to a multiplicative link: the factor chosen was ½. This second type of modeling, where the factor ½ of *q*^2^ of the first modeling approach is replaced by 2*q* (1 – *q*), is characterized by the dashed brown curve in Figure 1B. Like the first type of modeling, *q*^2^/2, this second type converts *q*^2^ into a function that never reaches unity, thereby taking into account negative cooperativity. The sum of 2*q* (1 – *q*) (black curve) and of *q*^2^ · 2*q*(1 – *q*) yields the solid brown curve in Figure 1B. We may learn again from this curve that at agonist concentrations of 0.032 μM and higher, more dimers with ultimately two of two β_1_-ARs occupied indicate a higher inotropic response despite the presence of the antagonist, as was the case with the solid blue curve of Figure 1B. The < signs in Table 1c correspond to this “increase in inotropy”: A pure β_1_-AR antagonist may enhance the inotropic action of endogenous multiple < signs β_1_-AR agonists at clinically relevant concentrations above their *K*_D_ (10^−8^ M = 0.01 μM). The solid blue and brown arrows on the right of the respective curve maxima in Figure 1B indicate the “increases in inotropy” due to the presence of a β_1_-blocker at low concentration.

The maximum of the solid brown curve is at about *q* = 0.61, which means that the agonist concentration at this maximum is 1.56 ^*^ [*K*_D_] (*q* = 0.61 = $\frac{\left[0.0156\right]}{\left[0.01]+[0.0156\right]}$). At higher agonist concentrations than 1.56 ^*^ [*K*_D_], the inotropic efficiency decreases again. Then, the added β_1_-blocker, by reducing $\frac{\left[L\right]}{K+\left[L\right]}$, shifts *q* to the left. As long as this left shift does not overcome the maximum of the brown curve at *q* = 0.61, the β_1_-AR antagonist yields a positive inotropic effect of the endogenous agonists. Antagonist-induced left shifts of *q* to values <0.61 may again worsen the positive inotropic condition: Too high β_1_-AR antagonist concentrations are known to worsen chronic heart failure, which fits the prediction of our modeling approaches.

Both types of modeling suggest that in CHF (with a concentration of endogenous agonist slightly or markedly higher than its *K*_D_), increased inotropic actions occur despite the presence of a low concentration of a pure β_1_-AR antagonist—a phenomenon related to the reduction of negative cooperativity. This is also true if inverse agonists (e.g., carvedilol), instead of pure antagonists, prevent negative cooperativity by occupation of one dimer receptor, leaving the other open for the endogenous agonist. Note that this negative cooperativity plays hardly any role at low agonist concentrations since then the occupation of two of two dimer receptors is rather unlikely.

Our modeling approaches neither assume that agonist binding to one dimeric receptor reduces the affinity of the second receptor for the antagonists nor that antagonist binding itself induces negative cooperativity. Albizu et al. (37) only observed negative cooperativity with agonists, not with antagonists.

As is known, a pure antagonist only binds to a receptor, but does not induce any receptor reaction. Strictly speaking, a reaction of antagonist binding, such as binding of this ligand to the first protomer decreasing the affinity of the same or another ligand for the second one, seems to be a contradiction in itself. Nevertheless, β_1_-adrenoceptor antagonists used to treat CHF may display some agonist properties and, therefore, also can possibly induce negative cooperativity. If such an “impure” antagonist binds to one dimeric β_1_-adrenoceptor and, therefore, the agonist binding to the other dimeric β_1_-adrenoceptor is reduced, then the term q = $\frac{\left[L\right]}{K+\left[L\right]}$ is changed to $\frac{\left[L\right]}{g\cdot K+\left[L\right]}$ with “*g*” >1. In other words, when *g* increases, *q* decreases. Then, the probability *q* of agonist binding on the x-axis in Figure 1B is shifted to the left: With higher agonist concentrations, the same condition as depicted in Figure 1B will be reached. Other sequels of our proposed modeling approaches do not have to be expected.

Correspondingly, when agonist binding to one dimeric receptor reduces the affinity of the second receptor for the antagonist, then the term $q=\frac{\left[endogenous\;agonist\right]}{\;K_{D}\;+\;10^{Lg\left[endogenous\;agonist\right]+Lg\left[K_{D}\right]-Lg\left[K_{A}\right]}\;+\;\left[endogenous\;agonist\right]}\;$ changes to $q=\frac{\left[endogenous\;agonist\right]}{\;K_{D}\;+\;10^{Lg\left[endogenous\;agonist\right]+Lg\left[K_{D}\right]-l\cdot Lg\left[K_{A}\right]}\;+\;\left[endogenous\;agonist\right]}\;$ with “*l*” < 1, but still *l* > 0. In other words, when *l* decreases, *q* slightly increases. Then, the probability *q* of agonist binding on the x-axis in Figure 1B is slightly shifted to the right: With a slightly higher antagonist concentration, the same condition as depicted in Figure 1B will be reached, again without any other sequels of our proposed modeling approaches.

## Conclusion

This article explains that, despite the presence of a low concentration of a β_1_-AR antagonist, a positive inotropic effect may occur in CHF. Our approach considers well-established prerequisites, i.e., (i) that the β_1_-ARs are spare (18–20), (ii) dimer receptors with activation of one receptor dimer already leading to the maximum effect (25), and (iii) that the concentration of the endogenous agonist (NA plus A) at the β_1_-AR is higher than its *K*_D_ value (21–23). Our calculation is based on the binomial distribution and shows that, due to the negative cooperativity of the receptor dimers (25), negative inotropy is converted to positive inotropy at moderate and high concentrations of the endogenous agonist.

The following questions remain. First, both proposed modeling approaches suggest a reduction in positive inotropy again if the concentration of the β_1_-adrenoceptor antagonist is so high that it shifts *q* too far to the left. Then *q* is in the ascending part of the solid blue or brown curve of Figure 1B. Does this condition with decreasing benefit point to the clinical observation that too high concentrations of β_1_-adrenoceptor antagonists can worsen chronic heart failure? Second, can increased and possibly harmful concentrations of β_1_-adrenoceptor antagonists be estimated accurately enough on the basis of the proposed modeling approaches to avoid clinical deteriorations in patients with chronic heart failure?

Nevertheless, this article may help to eliminate the skepticism of clinicians about the use of β_1_-blockers because of their supposed negative inotropic effect, since on the contrary, a positive inotropic effect can be expected for receptor-theoretical reasons. In addition, in the future, increased, and possibly harmful, concentrations of β_1_-blockers leading to clinical deterioration in patients with CHF may be avoided on the basis of the proposed modeling approaches.

## Data Availability Statement

The original contributions presented in the study are included in the article/supplementary material, further inquiries can be directed to the corresponding author.

## Author Contributions

All authors listed have made a substantial, direct and intellectual contribution to the work, and approved it for publication.

## Conflict of Interest

The authors declare that the research was conducted in the absence of any commercial or financial relationships that could be construed as a potential conflict of interest.
